# Delivering a research‐enabled multistakeholder partnership for enhanced patient care at a population level: The Northern Ireland Comprehensive Cancer Program

**DOI:** 10.1002/cncr.29814

**Published:** 2015-12-22

**Authors:** Mark Lawler, Anna Gavin, Manuel Salto‐Tellez, Richard D. Kennedy, Sandra Van Schaeybroeck, Richard H. Wilson, Denis Paul Harkin, Margaret Grayson, Ruth E. Boyd, Peter W. Hamilton, Darragh G. McArt, Jacqueline James, Tracy Robson, Robert D. Ladner, Kevin M. Prise, Joe M. O'Sullivan, Timothy Harrison, Liam Murray, Patrick G. Johnston, David J. Waugh

**Affiliations:** ^1^ Centre for Cancer Research and Cell BiologyQueen's University BelfastBelfastUnited Kingdom; ^2^ Northern Ireland Cancer Registry, Centre for Public HealthQueen's University BelfastBelfastUnited Kingdom; ^3^ Northern Ireland Molecular Pathology Laboratory, Centre for Cancer Research and Cell BiologyQueen's University BelfastBelfastUnited Kingdom; ^4^ Almac DiagnosticsCraigavonUnited Kingdom; ^5^ Northern Ireland Cancer CentreBelfastUnited Kingdom; ^6^ Northern Ireland Cancer Research Consumer ForumBelfastUnited Kingdom; ^7^ PathXL, Innovation Centre, Northern Ireland Science ParkBelfastUnited Kingdom; ^8^ Northern Ireland Biobank, Centre for Cancer Research and Cell BiologyQueen's University BelfastBelfastUnited Kingdom; ^9^ School of PharmacyQueen's University BelfastBelfastUnited Kingdom; ^10^ CV6 Therapeutics, Centre for Cancer Research and Cell BiologyQueen's University BelfastBelfastUnited Kingdom; ^11^ Almac DiscoveryCraigavonUnited Kingdom; ^12^ Centre for Public Health, Queen's University BelfastBelfastUnited Kingdom

## Abstract

The last 20 years have seen significant advances in cancer care in Northern Ireland, leading to measureable improvements in patient outcomes. Crucial to this transformation has been an ethos that recognizes the primacy role of research in effecting heath care change. The authors' model of a cross‐sectoral partnership that unites patients, scientists, health care professionals, hospital trusts, bioindustry, and government agencies can be truly transformative, empowering tripartite clinical‐academic‐industry efforts that have already yielded significant benefit and will continue to inform strategy and its implementation going forward.

## INTRODUCTION

*“This Centre is forging global partnerships to relieve the human suffering from cancer.”*

The words of former US Senator George Mitchell, captured on a giant 10‐meter high banner, greet every visitor to the Centre for Cancer Research and Cell Biology (CCRCB) at Queen's University Belfast (QUB) in Northern Ireland (NI), reflecting the aspiration to match local ambition with global collaboration to achieve better outcomes for patients with cancer. The opening of the CCRCB by Senator Mitchell represented an important step in a capacity‐building initiative that commenced in the mid‐1990s to address the fragmented and uncoordinated nature of cancer care and research in NI.

## THE CHALLENGE: FRAGMENTED CANCER SERVICES AND POOR CANCER OUTCOMES

The publication of the Campbell Report in 1996^1^ highlighted significant deficiencies in cancer services in NI. For example, among patients with breast cancer, there was wide variation in treatment provision across the province. Although some patients received quality care from specialist breast cancer teams, audit data indicated that 64% of surgeons in NI had performed <10 breast cancer surgeries per year.^1^ Similar issues were identified for several other malignancies, such that NI had the poorest cancer outcomes in the United Kingdom for the majority of cancers.^2^ However, as well as highlighting the problems that an uncoordinated cancer care system with a limited and underresourced cancer research activity can pose, the Campbell Report^1^ also provided a blueprint for change, recommending the development and implementation of a population‐level cancer plan that emphasized the need for a comprehensive and coordinated research‐enabled partnership approach to achieve improved outcomes for patients with cancer. Key principles of this strategy included: 1) the consolidation and expansion of cancer services within an overarching cancer center framework; 2) the production of comprehensive incidence and mortality data through a research‐active national cancer registry; 3) the introduction of robust cancer screening services; and 4) the development of an expanded research capacity to enable discovery science to be translated into clinical applications.

## RESPONDING TO THE CHALLENGE: EXEMPLARS OF AN INTEGRATED PARTNERSHIP‐ENABLED APPROACH TO IMPROVING CANCER CARE AND RESEARCH

The question remains: were the ambitions of the Campbell Report rewarded with measurable outputs of success? In the clinical setting, the introduction of a province‐wide 2‐view mammography screening program (unlike the single‐view approach that was used initially in the rest of the United Kingdom) raised attendance levels at breast screening clinics from just under 30% to nearly 80% and resulted in better detection of small invasive cancers. Breast cancer services were reorganized to ensure patients were seen by specialist teams with dedicated breast cancer surgeons rather than general surgeons with low‐volume activity as had been the case previously. Enhanced screening, in combination with this consolidation of breast cancer services, yielded a decrease in breast cancer mortality of 29.6% by 2006.^3^ Continuing this upward trend, by 2013, survival in NI for breast cancer was the highest in the United Kingdom (81.9%) compared with England (79.3%), Scotland (78.5%), and Wales (78.2%).^4^

From a public health perspective, the ability to capture NI population‐level data through the precise development and interrogation of comprehensive population‐based cohorts, particularly in patients with cancers of the gastrointestinal tract, has yielded findings that not only increased our understanding of disease etiology but also delivered population‐relevant, practice‐changing clinical interventions. The NI Barrett Esophagus Register^5^ is to our knowledge one of the largest population‐based registries of Barrett esophagus (BE) in the world (>13,000), and includes every patient in NI diagnosed between 1993 and 2010. The precise evaluation of this unrivalled resource has revealed that progression from BE to esophageal cancer is lower than was previously suspected,^6^ thus questioning the cost‐effectiveness of a BE surveillance program. Research on the NI BE cohort has also revealed that smoking leads to a 2‐fold increase in the risk of esophageal cancer,^7^ emphasizing the need for smoking cessation strategies for patients with BE. The availability of population‐based cohorts for colorectal adenoma/colorectal cancer (CRC) has also contributed to research with potentially practice‐changing clinical implications.^8^ Pharmacoepidemiological studies of general practitioner databases have shown that bisphosphonates (which are increasingly used to treat osteoporosis but are potentially associated with a higher risk of esophagitis) do not lead to an increased risk of esophageal cancer,^9^ thus supporting their continued use for the treatment of a common complication, particularly in postmenopausal women. Similar investigations of the use of statins after diagnosis have indicated that these drugs may increase overall survival in patients with CRC.^10^

From the perspective of translational research, biomarker and drug discovery research has underpinned the development of new prognostic/predictive tests and the identification of novel agents with therapeutic potential. For example, in CRC, gene expression profiling in >500 patients with AJCC TNM stage II disease (using proprietary CRC disease‐specific array technology developed in collaboration with Almac Diagnostics [Craigavon, UK]) has led to the identification of a 634‐gene signature (ColDx). Initial validation of this signature in primary tumors from 215 patients with AJCC TNM stage II disease from 12 centers as part of an international clinical trial yielded positive results that outperformed conventional clinicopathological risk factors (hazard ratio of 2.53 [95% confidence interval,1.54‐4.15] [*P* = .0003] for 5‐year recurrence‐free survival and 2.21 [*P* = .008] for cancer‐related death).^11^ This research has resulted in the development, commercialization, and licensing of a molecular test (GeneFx Colon; Helomics, Pittsburgh, Pa) with significant prognostic potential.^12^

These exemplars highlight the relevance of a cross‐sectoral, patient‐focused partnership (involving clinicians, allied health care professionals, pathologists, patient advocates, basic and translational scientists, bioinformaticians, biostatisticians, and the biotech/biopharma industry) to improve patient outcomes. The success of this partnership was recognized in 2012, when Queen Elizabeth II presented her Queen's Diamond Jubilee Award to the Vice Chancellor and the Dean of Medicine of QUB, thus valorizing the impact of the university‐led comprehensive cancer program on patient care.^13^

## COMPREHENSIVE CANCER CARE IN NI: PARTNERSHIP AND INTEGRATION

The development of an integrated cancer plan, aggregating services and expertise around a single comprehensive cancer center,^1^ and the implementation of a collaborative translational research strategy that values innovation and cross‐sectoral partnership have, as outlined above, underpinned a significant change in the last 20 years. The cross‐sectoral embrace of the program is key to its ongoing activities, connecting patient research advocacy, academic endeavor, industry rigor, and clinical excellence in an added‐value partnership (underpinned by support from government agencies, industry and academic leaders, National Health Service Hospital Trusts, research councils, and charities) that drives a translational continuum from discovery science to clinical application. Herein, we detail the collaborative activities of the key stakeholders that catalyzed and delivered a truly transformative change for patients with cancer in NI.

### Cancer at the Population Level: The Northern Ireland Cancer Registry

Having a functional, research‐enabled cancer registry that captures population‐level data has played a key role in the development of the comprehensive cancer program in NI. The Northern Ireland Cancer Registry (NICR) (www.qub.ac.uk/research-centers/nicr/) was founded in 1994 and demonstrates the value of an intersectoral partnership located within the Centre for Public Health at QUB, but funded through NI's Public Health Agency, a government body. Capturing cancer incidence, prevalence, and survival data at an entire population level is a vital resource, underpinning not only cancer research efforts but also service planning and cancer policy initiatives. Unlike many other European registries in which data coverage can be suboptimal (varying from 17%‐100%, with a European average of 50%),^14^ data coverage in the NICR is extremely high, with a registry completeness of 100%.^15^ In 1995, the NICR established what to our knowledge is the first entirely electronic total population‐based cancer registry in the world. The NICR embraces the outreach ethos that Senator Mitchell highlighted, contributing significantly to European and international initiatives including EUROCARE (a collaborative research project on cancer survivorship in Europe), the International Cancer Benchmarking Partnership (ICBP), and RARECARENet (an information network on rare cancers in Europe).^3^, ^4^, ^16^ An added‐value aspect of the NICR is its ability to collect data across the entire cancer journey because health and social care in NI are linked at a service delivery level, thus increasing the potential impact for both patients with cancer and cancer survivors. The NICR is increasingly patient‐focused and has recently commenced a £2.1‐million ($3.23‐million) Movember‐funded program to evaluate patient‐reported outcomes in individuals with prostate cancer.

### Molecular Pathology: The Foundation of Innovation

The establishment of the Northern Ireland Molecular Pathology Laboratory^17^ and the Northern Ireland Biobank (www.nibiobank.org) have been key infrastructural components of the translational research approach, providing a tripartite linkage between QUB, the hospital trusts, and the bioindustry sector. Collecting high‐quality, clinically annotated biospecimens, both for specific targeted cohorts and on a population basis through the Northern Ireland Biobank, has provided additional impetus to the translational research effort, delivering a rich resource with which to maximize the clinical impact of discovery science in molecular diagnostics, molecular therapeutics, and molecular cancer epidemiology.

Molecular pathology is a key enabler of the precision cancer medicine revolution, accelerating the introduction of new therapies through the discovery, validation, and clinical application of biomarkers for patient stratification and the evaluation of disease response. We have developed a hybrid molecular pathology model, moving away from the traditional division between diagnostics and translational research. In this model,^18^ we apply the same analytical, quality‐control, and organizational rigor to research discovery and its translation as we do to pathology test validation and its clinical application. The capability to perform both diagnostic and research functions is housed within a single “hybrid” laboratory infrastructure, with appropriate laboratory accreditation, thus ensuring the robust delivery of both the diagnostic requirements and the research translation activities of the program.

**Figure 1:**
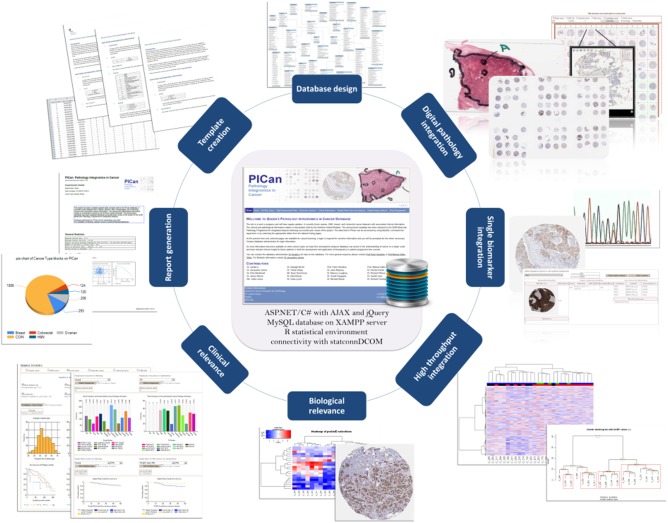
The PICan (Pathology Integromics in Cancer Platform) translational platform.

Within this integrated environment, we have also embedded digital pathology and automated quantitative imaging of tissue biomarkers,^19^ providing a platform for accelerated discovery, validation, and translation in patients with solid tumors. To realize the value of the comprehensive “omic” interrogation of cancer biospecimens, we developed a novel translational platform, PICan (Pathology Integromics in Cancer) (Fig. 1),^20^ that integrates all laboratory data (traditional, molecular, and digital pathology) with relevant clinical information, thus maximizing our ability to discover new biomarkers/therapeutic targets. PICan's integromics framework was tested and valorized in a study of a 288 tissue microarray cores from a cohort of patients with breast cancer, incorporating image‐related information including p53 immunohistochemistry and tissue pathology, mutational analysis, next‐generation sequencing, and unsupervised clustering of gene expression profiling, in combination with clinical data to comprehensively probe the involvement of p53 in breast cancer.^20^ PICan's browser‐based approach allows for data analysis and interpretation and digital image assessment to be achieved seamlessly, facilitating the incorporation of multiple high‐dimensional data into a single searchable interface. The translational bioinformatics team continues to build on this unique platform for internal tissue biomarker discovery and validation of multiplex genomic signatures.

Moving forward, a QUB‐led collaborative approach with centers at the University of Southampton, University College London, University of Manchester, University of Newcastle, and University of Leicester that was recently supported through 1 of 5 Cancer Research UK (CRUK) Centres Network Accelerator Awards^21^ will allow this integrated digital and molecular pathology model to be standardized and adopted at a UK level.

### Bringing Precision Medicine to the Clinic: The NI Cancer Trials Centre and the Experimental Cancer Medicine Centre

In 1999, a historic agreement was signed between the US National Cancer Institute (NCI) and the departments of health in both the Republic of Ireland and NI. The NCI‐Ireland‐Northern Ireland Memorandum of Understanding underpinned the development of appropriate infrastructure in cancer epidemiology, cancer care, and cancer research and education on the island of Ireland^22^ and its outputs and legacy are the subject of a separate publication (unpublished data). From the perspective of clinical cancer research and cancer treatment, one of the first key outcomes from this agreement was the establishment of a robust cancer clinical trials infrastructure through the creation of the Northern Ireland Cancer Clinical Trials Unit (now known as the Northern Ireland Cancer Trials Centre). Since its establishment in early 2000, the Northern Ireland Cancer Trials Centre has significantly enhanced participation in cancer clinical trials in Northern Ireland, such that in the period between April 2013 and March 2014, a total of 1665 patients in Northern Ireland were recruited to cancer clinical trials and related translational studies, a rate that is nearly twice the recruitment target of 850 patients (10% of the incident cancers in NI).^23^

This enhanced cancer clinical trial activity, underpinned by patient‐focused discovery research and its translation, led to the subsequent designation of the CCRCB as a CRUK center (one of the first to be established in the United Kingdom) in 2009 and a Department of Health/CRUK Experimental Cancer Medicine Centre (ECMC) in 2007. Focused on driving early‐phase clinical trials, the ECMC provides the perfect conduit for translating discovery science conducted within the research program into new treatments for patients with cancer.

A key exemplar of this approach has resulted from a discovery joint research program between QUB, Almac Discovery, and Almac Diagnostics. Discovery research revealed that the endogenous protein FKBPL (FK506 binding‐like) has significant antiangiogenic properties and is mediated through a CD44‐dependent mechanism.^24^, ^25^ In collaboration with Almac Discovery, a 23‐amino acid linear peptide that represents the active domain of FKBPL has been developed (ALM201). QUB is now leading a national first‐in‐human phase 1 clinical trial of this novel antiangiogenic peptide, ALM201,^26^ for the treatment of platinum‐resistant ovarian cancer (EudraCT number: 2014‐001175‐31). This trial is now actively recruiting patients across 3 ECMC locations in the United Kingdom and will be biomarker‐driven, using a novel “angiogenic (AADX) signature” developed by Almac Diagnostics and Edinburgh University to select patients for the expansion phase of the trial.

In patients with CRC, research within the center concerning drug‐resistant signaling pathways implicated activation of c‐MET as a key mediator of resistance to MEK inhibition in patients with *KRAS‐*mutant CRC,^27^ providing the rationale for a successful €6‐million (approximately $6.65 million) European Commission Framework VII‐funded bid for the CCRCB to lead a pan‐European translational clinical trial program with 13 partners from 7 European countries (MErCuRIC trial) (mercuric.eu/). The novel therapeutic strategy of the MErCuRIC trial investigates the combination of a MET and a MEK inhibitor to combat metastasis, improve survival, and potentially develop a new clinical intervention approach in patients with this poor‐prognosis subtype of CRC.

In patients with prostate cancer, QUB, in partnership with the University of Manchester, has been awarded a £5‐million (approximately $7.8 million) program to establish FASTMAN (BelFAST MANchester), an International Movember Centre of Excellence in Prostate Cancer Research^28^ to address the issue of disease recurrence and the use of advanced radiotherapy and DNA damage‐inducing treatments in this disease. All these examples emphasize how complimentary partnerships with academia and/or bioindustry are driving our precision medicine pipeline, and delivering novel and informed opportunities to benefit patients.

### Crossing the Rubicon: Bridging the Academia‐Industry Divide

The significant resources invested by the pharmaceutical industry to create “in‐house” oncology research and development capacity has yielded limited success rates for new drug approvals, and therapeutic improvements that can often only be considered as marginal.^29^, ^30^ Within the CCRCB, our investigators have been quick to adopt a team science approach, but also to realize that team science is not the sole preserve of academia and also should straddle the academia‐industry intersect for mutual advantage and benefit to patients. In pooling resources and expertise, we have promoted the belief that we derisk biomarker and drug discovery/development programs, allowing for the delivery of transformative solutions that will improve patient outcomes. Assets such as the antiangiogenic peptide ALM201^26^ and the GeneFX Colon prognostic/predictive test^12^ stemming from our collaborative pipeline as described above are now beginning to demonstrate the validity and merits of such an approach.

### CCRCB‐Almac: A Mutually Productive Academia‐Industry Partnership

Recognizing the need to bridge the divide outlined above,^31^ the CCRCB has promoted increased engagement between academia and industry. Almac is one of NI's leading indigenous companies, operating in the pharmaceutical and biotechnology sectors and employing >3500 employees worldwide. Within the Almac Group, Almac Diagnostics specializes in biomarker discovery and development. It has historical linkages with QUB, spinning out from the CCRCB in the early 2000s. The recent collaborative partnership, in operation since 2010, has been extremely productive, delivering a series of high‐impact publications^11^, ^32^, ^33^ and several validated clinical tests, as well as licensing and collaborative agreements with global biotechnology/biopharmaceutical companies. For example, discovery DNA microarray analysis of the Fanconi anemia/BRCA DNA damage response (DDR) pathway identified a 44‐gene expression‐based DDR deficiency signature.^32^ Evaluation of this 44‐gene signature in 3 independent data sets from 203 patients with breast cancer highlighted the ability of this biology to predict response to DNA‐damaging chemotherapy, leading to the development of a formalin‐fixed paraffin‐embedded tissue assay that was validated as a predictor of response in the neoadjuvant setting and recurrence‐free survival in the adjuvant setting after anthracycline/cyclophosphamide‐based therapy.^32^

CCRCB researchers, in collaboration with Almac Diagnostics, also have identified the potential for this biomarker to be relevant in patients with CRC, and this work has led to the CCRCB becoming a significant partner in a £5‐million (approximately $7.8 million) University of Oxford‐led Medical Research Council‐CRUK stratified medicine program in CRC (S‐CORT [Stratification in COloRecTal cancer]),^34^ which brings together all the major UK centers in CRC research in a national collaborative partnership with bioindustry and patient advocacy groups. S‐CORT aims to develop novel validated biomarker tests to stratify patients for more effective chemotherapy‐based, radiotherapy‐based, or surgical or novel biological therapeutic interventions.^35^

Key joint academic industry appointments at the professorial level have helped to embed the QUB‐Almac partnership approach for mutual benefit. We have nurtured this close relationship, creating a “spin‐in” biomarker and drug discovery research hub, with >25 research scientists from Almac currently embedded within the CCRCB, operating in close proximity to the academic research programs. There have been tangible benefits to QUB, not least the adoption of an industry rigor to our core operation and research practice, together with an appreciably greater entrepreneurial culture permeating across the scientific and clinical staff. The physical integration of the collaborating research structures with their complementary skill sets has led to a productive acceleration of asset delivery within the biomarker and drug development pipelines as outlined above, and the industrial partner has recognized the value of direct access to the clinical expertise of the university/hospital to inform clinical development while exploiting the wider biomedical and medical expertise to appraise other potential exploitable medical indications for their products.

### Embedding National and International Joint Academia‐Industry Partnerships

A crucial partner in enabling our precision medicine program has been the local government development agency, Invest Northern Ireland, which has recognized the importance of academic‐industrial collaboration in expanding the life science sector and directly contributing to the growth of the regional knowledge‐based economy. The success of the CCRCB‐Almac partnership model has been adapted further and supported to help embed other joint academia‐industry activities within the CCRCB. Such examples follow below.

#### PathXL

PathXL (www.pathxl.com) is a digital pathology software company that spun out of the CCRCB and has become a global leader in the provision of innovative digital pathology solutions for biomarker/drug discovery research and clinical applications. The company, in partnership with the CCRCB, has pioneered a novel software algorithm (TissueMark) for automated tumor recognition and quantitative analysis for nucleic acid extraction and molecular diagnostics in solid tumors.^36^ TissueMark was tested and validated for its ability to provide automated tumor annotation and measurement of the percentage of tumor nuclei in 245 hematoxylin and eosin‐stained slides from patients with non‐small cell lung cancer. The automated approach demonstrated strong concordance with manually drawn boundaries and automated analysis of cell counts for percentage tumor measurements by TissueMark demonstrated reduced variability and significant correlation (*P*<.001) with benchmark tumor cell counts.^36^ This technology is now being applied across lung, colon, breast, and prostate cancer, thereby facilitating tissue‐based molecular assays including next‐generation sequencing. These studies have demonstrated that robust image analysis technology can facilitate the automated quantitative analysis of tissue samples for molecular profiling, bridging the gap between traditional anatomical pathology and molecular diagnostics and providing the appropriate quality for application in precision medicine.

#### CV6 Therapeutics

Attracting international bioindustry into the program, based on the academia‐industry partnership model described above, also has been successful. CV6 Therapeutics has relocated from the University of Southern California and is now embedded within the CCRCB.^37^ Targeting a process such as thymidylate biosynthesis and the enzyme thymidylate synthase have been critical components of cancer treatment, with fluoropyrimidines such as 5‐fluorouracil and antifolates such as methotrexate underpinning therapeutic intervention in many solid tumors and hematological malignancies. Researchers at CV6 Therapeutics have characterized resistance mechanisms within the thymidylate biosynthesis pathway^38^ and are using this knowledge to drive the development of new innovative therapeutic approaches that will have relevance and clinical applicability for many cancers. Complementing existing research in drug resistance mechanisms (particularly in CRC) by CCRCB researchers,^27^, ^39^, ^40^, ^41^ this partnership has significant potential to deliver new drug combinations and novel approaches with therapeutic potential.

Overall, our partnership model has proven attractive to the biotechnology and industry sectors, allowing distinct but complementary skills to be brought together in an academic environment that favors innovation, with the ability to unlock new collaborative funding schemes at local, national, and international levels. The model also enhances the opportunities for testing potential industry assets in appropriate clinical settings. Similar cross‐sectoral approaches are being developed in this cancer drug discovery space between the University of Manchester, GlaxoSmithKline, and Cancer Research Technology, with a particular emphasis on epigenetic targeting in cancer,^42^ whereas Pfizer's Centers for Therapeutic Innovation are strategically placed in biomedical research hubs and aligned with academic centers in Boston, New York, San Diego, and San Francisco and have recently partnered with the National Institutes of Health's National Center for Advancing Translational Sciences.^43^

### Patient‐Focused Research: The Northern Ireland Cancer Research Consumer Forum

The patient voice is a key supporter of an evolving precision cancer medicine partnership to deliver personalized cancer care. The Northern Ireland Cancer Research Consumer Forum (NICRCF)^23^ engages at the earliest stages of the translational process, being involved in research grant preparation (the NICRCF is a funded partner in both the MErCuRIC and S‐CORT programs) and submission, clinical trial design, and patient‐reported outcomes research, thus providing a distinctive and extremely beneficial added‐value flavor to the research effort. Patients increasingly want to be part of the research process. A recent strategic review of personal and public involvement (PPI) in research (commissioned by the National Institute for Health Research) emphasizes the importance of a partnership model.^44^ In relation to cancer, a survey of 811 patients revealed that > 90% indicated a desire for their samples to be used in future research, whereas a significant number of patients also indicated that they wanted to participate in genomic testing to allow for personalization of their treatment.^45^ As a member of the European Patients' Academy on Therapeutic Innovation (EUPATI),^46^ patient advocates from the NICRCF promote a wider appreciation among patients and the general public of the benefits and challenges of translational research and its application.

Although patient advocacy groups are particularly active within the global cancer community, a PPI that focuses primarily on research is unusual, particularly in the European context. NICRCF members have a very particular experience of cancer, either as a cancer survivor, a member of a family affected by cancer, or as a cancer caregiver, and are committed to work with cancer researchers and health care professionals to advance cancer research activities and initiatives. In collaboration with Macmillan Cancer Support, the NICRCF has piloted “Building Research Partnerships in Northern Ireland,” a shared learning activity between patients and health care professionals to support PPI in health and social research. Semiquantitative assessment of stage I (immediate evaluation) has indicated > 95% efficacy in meeting participants' needs, whereas stage II of the activity (transfer of learning evaluation) is ongoing and its results are eagerly awaited. As part of the S‐CORT program, we also will attempt to measure the value that patient advocacy brings to research endeavors through a combination of surveys and qualitative/semiquantitative assessments. The NICRCF combines a pro‐research advocacy agenda with a gatekeeper function to ensure that the best, most ethically responsible research is conducted that delivers both academic excellence and patient relevance.

### Education and Outreach: Innovative Doctoral Training and Public Engagement

At a clinical level, a clinical academic training program, mediated initially through fellowship training and short‐stay visits at the NCI and at NCI comprehensive cancer centers as part of the All‐Ireland‐NCI Cancer Consortium, underpinned the development of a cohort of individuals who have now assumed leadership roles in clinical oncology, radiation oncology, and cancer clinical trials. Specialist nursing expertise, particularly for cancer clinical trials, also was developed through this mechanism.

Underpinning the framework described above are a series of innovative educational and outreach activities. The CCRCB doctoral training program in precision cancer medicine is providing students not only with the scientific rigor and wet laboratory/bioinformatics skill sets with which to conduct high‐quality research, but also with the business, entrepreneurial, innovation, and leadership skills and acumen that will benefit them in their evolving careers. Embedding bioindustry, the QUB's Management School, and the William J. Clinton Leadership Institute within the educational program is ensuring the active transfer of relevant career‐enhancing skills to the students. A new aspect of the program is a recently established partnership with the NCI in the United States, allowing students to perform research at the NCI as part of their PhD studies. In this program, students first complete a Masters by Research degree in precision cancer medicine, acquiring not only the technical laboratory expertise but also the innovation, business, and leadership skills to underpin their future careers. Subsequent mentorship at the NCI as part of the NIH Graduate Partnership Program will provide the students with a comprehensive research training experience.

In pathology, we recognize the need for histopathologists to undergo molecular pathology training^47^ and have developed a model for integrating specialist formal training in molecular diagnostic/molecular pathology within the existing 5‐year histopathology training curriculum.^48^ The recent CRUK Accelerator Award is underpinning this innovative training program through a blended learning Masters in the Molecular Pathology of Cancer degree delivered by QUB for pathology trainees in Belfast, the University of Southampton, University College London, the University of Manchester, the University of Newcastle, and the University of Leicester.

From a public engagement perspective, a vibrant outreach program, supported through Cancer Research UK and local charities and delivered by our Engagement Committee, highlights our precision medicine approach to personalized cancer care. Precision medicine has been a significant theme at the inaugural NI Science Festival, the CCRCB Open Day, and a recent Culture Night event. These engagement events provide an essential opportunity to inform and educate our stakeholders of the importance of research to improving patient care and outcomes. At a political level, we deliver engagement activities at the NI Parliament buildings and regularly host government and all‐party committees at the CCRCB, thus both informing politicians and policy makers of the importance of research as well as highlighting the shared responsibilities for all stakeholders to enhance the health and wealth of NI's citizens and societies.

## IMPLEMENTING OUR VISION THROUGH CONTINUED PATIENT‐FOCUSED AND RESEARCH‐ENABLED INTERSECTORAL PARTNERSHIP

As we have demonstrated in this article, the last 20 years have witnessed significant advances in cancer care in NI, leading to measureable improvements in patient outcomes. Crucial to this transformation has been an ethos that recognizes the primacy role of research in effecting heath care change. Our model of a cross‐sectoral partnership that unites patients, scientists, health care professionals, hospital trusts, bioindustry, and government agencies can be truly transformative, empowering tripartite clinical‐academic‐industry efforts that have already yielded significant benefit and will continue to inform our strategy and its implementation going forward. NI has a population of only 1.85 million individuals, with approximately 9000 new patients diagnosed with cancer each year (excluding nonmelanoma skin cancer), but it is precisely this compact size that works in our favor, allowing cancer research and cancer services to be integrated, planned, and delivered at the population level. A coordinated partnership of all the key stakeholders with a clear unity of purpose is beginning to catalyze a truly integrated research‐driven comprehensive cancer program, which we will continue to deliver through a linked health and social care infrastructure that allows change to be effected across the cancer continuum. As we move forward, the CCRCB hopes to attract other biotechnology and pharma partners to exploit the established discovery pipeline. Expanded access will deliver significant health, wealth, and societal benefits to NI and beyond, but also keep faith with the challenge laid down by Senator Mitchell, to use “global partnerships to relieve human suffering from cancer.”

## FUNDING SUPPORT

Supported by Cancer Research UK, Medical Research Council UK, Prostate Cancer UK, European Commission Framework VII, and Invest Northern Ireland.

## CONFLICT OF INTEREST DISCLOSURES

Manuel Salto‐Tellez has received personal fees from Ventana, Roche, Pfizer, PathXL, Almac, and AstraZeneca for work performed outside of the current study. Richard D. Kennedy is the paid medical director for Almac Diagnostics and is an author of issued patent WO/2012/037378 (DNA damage response deficiency signature assay from Almac). Richard H. Wilson has received grants from Cancer Research UK, Northern Ireland HSC Research and Development Division, and Friends of the Cancer Centre for work performed as part of the current study and has received honoraria from and had advisory roles with Roche, Hospira, Sanofi Oncology, and Merck Serono for work performed outside of the current study. Denis Paul Harkin is the Chief Executive Officer of Almac Diagnostics. Peter W. Hamilton has received grants from Cancer Research UK, Invest Northern Ireland, Marie Curie FP7 EU, and HSC Research and Development Agency for work performed as part of the current study; has received outside fees as the founder of, shareholder in, and director of PathXL Ltd and Lewis Fertility Tests Ltd; and has the following patents pending: UK App No. 1315597.3, UK App No. 1509878.3, UK App No. 1308460.3, UK App No. 1509886.6, UK App No. 1516866.9, UK App No. 1516869.3, UK App No. 1516873.5, UK App No. 1516871.9, PCT/GB2014/051427, and PCT/GB2014/051426. Tracy Robson has received a grant from Almac Discovery for work performed as part of the current study and has patent WO/2007/141533 issued and licensed and patent WO/2010/133880 issued. Robert D. Ladner is the Chief Executive Officer of and a shareholder in CV6 Therapeutics and has a pending and licensed patent for Deoxyuridine Triphosphate Inhibitors for CV6 Therapeutics. Timothy Harrison is an employee of Queen's University Belfast and is Vice President of Almac Discovery. Patrick G. Johnston is the previous founder of and a shareholder in Almac Diagnostics; has received honorarium and acted as a paid consultant for Pfizer and Chugai Pharmaceuticals for work performed outside of the current study; and has stock ownership and interest in Almac Diagnostics, Fusion Antibodies, and CV6 Therapeutics. David J. Waugh has received grants from HPSS Research and Development Division, Medical Research Council, Cancer Research UK, and Movember/Prostate Cancer UK for work performed as part of the current study and has acted as a paid consultant and member of the Advisory Board for Almac Group and Almac Discovery.
